# Discovery and characterization of a small‐molecule enteropeptidase inhibitor, SCO‐792

**DOI:** 10.1002/prp2.517

**Published:** 2019-09-04

**Authors:** Masako Sasaki, Ikuo Miyahisa, Sachiko Itono, Hiroaki Yashiro, Hideyuki Hiyoshi, Kazue Tsuchimori, Ken‐ichi Hamagami, Yusuke Moritoh, Masanori Watanabe, Kimio Tohyama, Minoru Sasaki, Jun‐ichi Sakamoto, Tomohiro Kawamoto

**Affiliations:** ^1^ Research Takeda Pharmaceutical Company Limited Fujisawa Kanagawa Japan; ^2^ SCOHIA PHARMA, Inc. Fujisawa Kanagawa Japan; ^3^ Present address: Axcelead Drug Discovery Partners, Inc. Fujisawa Kanagawa Japan

**Keywords:** covalent, enteropeptidase, FRET, SCO‐792

## Abstract

Enteropeptidase, localized into the duodenum brush border, is a key enzyme catalyzing the conversion of pancreatic trypsinogen proenzyme to active trypsin, thereby regulating protein digestion and energy homeostasis. We report the discovery and pharmacological profiles of SCO‐792, a novel inhibitor of enteropeptidase. A screen employing fluorescence resonance energy transfer was performed to identify enteropeptidase inhibitors. Inhibitory profiles were determined by in vitro assays. To evaluate the in vivo inhibitory effect on protein digestion, an oral protein challenge test was performed in rats. Our screen identified a series of enteropeptidase inhibitors, and compound optimization resulted in identification of SCO‐792, which inhibited enteropeptidase activity in vitro, with IC_50_ values of 4.6 and 5.4 nmol/L in rats and humans, respectively. In vitro inhibition of enteropeptidase by SCO‐792 was potentiated by increased incubation time, and the calculated *K*_inact_/K_I_ was 82 000/mol/L s. An in vitro dissociation assay showed that SCO‐792 had a dissociation half‐life of almost 14 hour, with a calculated *k*_off_ rate of 0.047/hour, which suggested that SCO‐792 is a reversible enteropeptidase inhibitor. In normal rats, a ≤4 hour prior oral dose of SCO‐792 effectively inhibited plasma elevation of branched‐chain amino acids in an oral protein challenge test, which indicated that SCO‐792 effectively inhibited protein digestion in vivo. In conclusion, our new screen system identified SCO‐792 as a potent and reversible inhibitor against enteropeptidase. SCO‐792 slowly dissociated from enteropeptidase in vitro and inhibited protein digestion in vivo. Further study using SCO‐792 could reveal the effects of inhibiting enteropeptidase on biological actions.

**Abbreviations**
BCAA: branched‐chain amino acids
FRET: Fluorescence resonance energy transfer
HTS: High‐throughput screening

## 1 INTRODUCTION

Proteins are pivotal macronutrients for various cellular activities, as well as whole‐body metabolism. Amino acids show many functions within the body. For example, amino acids serve as the only source of nitrogen in mammals.^1^ Amino acid‐derived nitrogen is a critical element in the synthesis of the precursors (purine and/or pyrimidine) of major energy molecules, such as adenosine triphosphate, adenosine diphosphate, and/or nucleic acids.^1^ In addition, nitrogen is incorporated into compounds that can regulate major biochemical signaling pathways, such as nitric oxide.^1^ Furthermore, amino acid deamination in the body's proteins generates a carbon skeleton rich in oxygen and hydrogen suitable for subsequent biochemical transformation.^1^ This carbon skeleton can be used by the liver to generate glucose through gluconeogenesis and other macromolecules, such as lipids.^1^ The carbon skeleton derived from amino acids is also relevant in producing intermediaries fueling the Kreb's cycle that are thereafter transformed into energy and/or other metabolic molecules.^1^ Taken together, amino acids can be considered as biochemical molecules that can be converted into energy, carbohydrates, lipids, and biochemical intermediates depending on the specific bodily metabolic situation.

Degradation of dietary proteins and subsequent amino acid absorption is a key step in maintaining protein homeostasis in mammals.^2^, ^3^, ^4^ Activation of pancreatic enzymes is essential for digestion of proteins to give amino acids that are subsequently absorbed in the gut. During food digestion in the gut, enteropeptidase (EC 3.4.21.9) serves as a critical upstream molecule in the process of protein digestion.^5^ Enteropeptidase is a serine protease localized on the intestinal brush border. The enzyme catalyzes the conversion of inactive trypsinogen, which is secreted from the pancreas into the gut, to active trypsin^6^. The activated trypsin in turn activates downstream digestive enzymes, such as chymotrypsinogen, proesterase, procarboxypeptidases A and B, and prolipase, which allow the absorption of amino acids and triglycerides in the gut.^5^ Congenital enteropeptidase deficiency in humans has resulted in intestinal malabsorption and a lean phenotype, which suggest the pivotal role of this enzyme in regulating body homeostasis.^7^, ^8^ Interestingly, a recent seminal study suggested that inhibiting gut enteropeptidase may be a novel strategy for correcting obesity.^9^ Concomitantly, accumulating reports suggest that the strong connection of plasma amino acid change with obesity and insulin resistance.^10^ Considering that enteropeptidase is an upstream key molecule in a protein degradation process,^5^ identifying potent and effective new enteropeptidase inhibitors and determining their biological actions in health and disease statuses are of great interest.

Thus, the current study was conducted to identify new enteropeptidase inhibitors and characterize their in vitro and in vivo biological activity profiles. We first constructed a new high‐throughput screening (HTS) system to identify compounds inhibiting enteropeptidase in vitro. After screening, an optimized compound, SCO‐792,^11^ was further characterized by in vitro and in vivo studies.

## 2 MATERIALS AND METHODS

### 2.1 Materials

SCO‐792 (N‐({(3S)‐6‐[(4‐carbamimidamidobenzoyl)oxy]‐2,3‐dihydro‐1‐benzofuran‐3‐yl}acetyl)‐L‐aspartic acid hydrate) was synthesized by Takeda Pharmaceutical Company Limited. The substrates QSY21‐Gly‐Asp‐Asp‐Asp‐Lys‐Ile‐Val‐Gly‐Gly‐Lys (Cy5) and 5FAM‐Abu‐Gly‐Asp‐Asp‐Asp‐Lys‐Ile‐Val‐Gly‐Gly‐Lys (CPQ2)‐Lys‐Lys‐NH_2_ were purchased from CPC Scientific (Sunnyvale, CA). H‐Gly‐Asp‐Asp‐Asp‐Asp‐Lys‐βNA was purchased from Bachem (Bubendorf, Switzerland). QXL520‐Gaba‐IHPFHLVIHTK (HiLyteFluo488) R and Boc‐Phe‐Ser‐Arg‐MCA were purchased from the Peptide Institute (Osaka, Japan). Human recombinant enteropeptidase was purchased from ITSI‐Biosciences (Johnstown, PA). Rat recombinant enteropeptidase was expressed in *Escherichia coli* BL21 (DE3) and purified by STI‐agarose. Human recombinant renin was purchased from Anaspec (Fremont, CA). Human trypsin, dimethyl sulfoxide (DMSO), bacterial leucine dehydrogenase, and L‐leucin were purchased from FUJIFILM Wako Pure Chemical Corporation (Osaka, Japan). Methylcellulose SM‐100 was purchased from Shin‐Etsu Chemical (Tokyo, Japan).

### 2.2 Enteropeptidase enzyme assay

In the HTS, enzyme and substrate were dissolved in the enteropeptidase assay buffer [50 mmol/L Tricine, pH 8.0, 0.01% (w/v) Tween20, and 10 mmol/L CaCl_2_]. Twenty‐five nanoliters of compound solution dissolved in DMSO was added to a 1536‐well black plate, and then 2 μL of 90 mU/mL human recombinant enteropeptidase solution was added to the plate and incubated at room temperature for 60 minutes. Next, 2 μL of substrate solution [2.1 μmol/L QSY21‐Gly‐Asp‐Asp‐Asp‐Lys‐Ile‐Val‐Gly‐Gly‐Lys (Cy5)] was added to the plate and incubated at room temperature for 30 minutes. After incubation, 2 μL of 30 mmol/L H_2_SO_4_ solution was added to stop the reaction. The fluorescence was measured at an excitation wavelength of 620 nm and an emission wavelength of 685 nm by multilabel plate reader EnVision (PerkinElmer, Waltham, MA).

For kinetic analysis, compounds were dissolved in DMSO and then diluted in the enteropeptidase assay buffer. Five microliters of compound solution was added to a 384‐well black plate followed by 5 μL of substrate solution [2.1 μmol/L 5FAM‐Abu‐Gly‐Asp‐Asp‐Asp‐Lys‐Ile‐Val‐Gly‐Gly‐Lys (CPQ2)‐Lys‐Lys‐NH_2_] and 5 μL of 24 mU/mL human recombinant enteropeptidase solution and mixed. The final concentration of substrate was 0.7 μmol/L, which is almost the same as the *K*_*m*_ value. The fluorescence was measured every minute at an excitation wavelength of 485 nm and an emission wavelength of 520 nm using an EnVision multilabel plate reader. The progress curves were fitted to the following equation to determine the values for *k*_obs_, the apparent rate constant from initial rate *v*_*0*_ to steady state rate *v*_*s*_:

(1) $$F=v_{s}t+\left(\left(v_{0}-v_{s}\right)\left(1-e^{-k_{\mathrm{obs}}t}\right)\right)/k_{\mathrm{obs}}+F_{0},$$

where *t* is the time, *F* is the fluorescence, and *F*_0_ is the fluorescence at time *t* = 0.

The *k*_inact_/*K*_*I*,app_ value was determined as the slope of the [I] vs *k*_obs_ plot, and the corrected *k*_inact_/*K*_*I*_ value was also estimated according to the following equation:

(2) $$k_{\mathrm{inact}}/K_{I,\mathrm{app}}=k_{\mathrm{inact}}/K_{I}/\left(1+\left[S\right]/K_{m}\right),$$

where [*I*] is the concentration of inhibitor, [*S*] is the concentration of substrate, and *K*_*m*_ is the Michaelis–Menten constant.

All enteropeptidase enzyme assay and compound evaluation were conducted at pH 8 because the optimal pH of enteropeptidase was 8 as previously reported^12^; Magee et al.^13^

### 2.3 Renin enzyme assay

Compounds were dissolved in DMSO and then diluted in renin assay buffer [20 mmol/L phosphate buffer, pH 7.4, 0.01% (w/v) Tween20]. Three microliters of compounds diluted in assay buffer was added to a 384‐well nonbinding surface black plate. Then, 3 μL of 150 ng/mL recombinant renin was added to the plate and incubated at room temperature for 60 minutes. After this incubation, 3 μL of 3 μmol/L substrate solution [QXL520‐Gaba‐IHPFHLVIHTK (HiLyteFluo488) R] was added to the plate. After incubation at room temperature for 60 minutes, the reaction was stopped by the addition of 3 μL of 80 mmol/L H_2_SO_4_. The fluorescence at an excitation wavelength of 485 nm and an emission wavelength of 535 nm was detected using an EnVision multilabel plate reader.

### 2.4 Trypsin enzyme assay

Compounds were dissolved in DMSO and then diluted in trypsin assay buffer [50 mmol/L Tris‐HCl, pH 7.5, 145 mmol/L NaCl, 2 mmol/L CaCl_2_, and 0.01% (w/v) Tween20]; then; 2 μL of compound solution was added to a 384‐well black plate. Next, 8 μL of substrate solution (31.25 μmol/L Boc‐Phe‐Ser‐Arg‐MCA) and 10 μL of 4 mU/mL human trypsin solution were added and incubated at room temperature for 60 minutes. The fluorescence at an excitation wavelength of 355 nm and an emission wavelength of 460 nm was detected using an EnVision multilabel plate reader.

### 2.5 Dissociation assay

For the dissociation assay, compounds were dissolved in DMSO and then diluted in the enteropeptidase assay buffer. Ten microliters of compound solution was added to a 96‐well plate, and then 10 μL of 100 mU/mL human recombinant enteropeptidase solution was added to the plate and incubated at room temperature for 120 minutes. The concentration of the compound was equal to 10‐fold of the IC_50_ value upon incubation for 120 minutes. After this incubation, 2 μL of an compound‐enzyme mixture was transferred to a 96‐well black plate, and then 200 μL of substrate solution [3 μmol/L 5FAM‐Abu‐Gly‐Asp‐Asp‐Asp‐Lys‐Ile‐Val‐Gly‐Gly‐Lys(CPQ2)‐Lys‐Lys‐NH_2_] was added to the well. By its rapid dilution, the concentration of the inhibitor dropped from 10‐fold above the IC_50_ to 10‐fold below it. The fluorescence was measured every 60 minutes at an excitation wavelength of 485 nm and an emission wavelength of 520 nm using an EnVision multilabel plate reader. The progress curves were fitted to the following equations to determine the values for *k*_off_ and dissociation half‐life, *t*_1/2_.

(3) $$F=v_{s}t+\left(\left(v_{0}-v_{s}\right)\left(1-e^{-k_{\mathrm{off}}t}\right)\right)/k_{\mathrm{off}}+F_{0}$$

(4) $$t_{1/2}=\text{ln}\left(2\right)/k_{\mathrm{off}}$$

### 2.6 Animals

All animals were housed in a room with controlled temperature (23°C), humidity (55%), and lighting (lights on between 7:00 am and 7:00 pm). All animals were allowed free access to standard laboratory chow diet (CE‐2; CLEA Japan, Inc.) and tap water. The care and use of animals and the experimental protocols were approved by the Experimental Animal Care and Use Committee of Takeda Pharmaceutical Company, Ltd. All experiments were performed according to the guidelines and regulations of the Takeda Pharmaceutical Company, Ltd., and Shonan Health Innovation Park. All blood samples used in the present study were obtained via the tail veins of the animals.

### 2.7 Pharmacokinetic study in rats

Male Sprague‐Dawley rats were obtained from Charles River Laboratories Japan, Inc. (Yokohama, Japan). A pharmacokinetic study was conducted when the animals were 8 weeks old. For oral administration, SCO‐792 was suspended in a 0.5% (w/v) methylcellulose solution. For intravenous administration, SCO‐792 was dissolved in DMSO and added with saline to prepare a dosing formulation at a concentration of 0.2 mg/mL (DMSO:saline = 2:8 (v/v)). The oral dosing formulations were mixed well and given to fed‐male rats at single doses of 10 mg/5 ml/kg using polypropylene syringes with gavage needles. The intravenous dosing formulation was injected into the femoral vein of fed‐male rats at a dose of 0.2 mg/1 ml/kg using polypropylene syringes with needles under anesthesia with isoflurane. The blood was collected at the indicated time points, and the plasma concentrations of SCO‐792 were determined by high‐performance liquid chromatography/tandem mass spectrometry.

### 2.8 Oral protein challenge in rats

Male Sprague‐Dawley rats were obtained from CLEA Japan Inc. (Tokyo, Japan). Eight‐week‐old rats were randomized into three groups on the basis of body weight and body weight change before the experiment (n = 4). SCO‐792 (10, 30 mg/kg in a 0.5% (w/v) methylcellulose solution) was then orally administered 1, 2, 4, and 6 hours before an oral whey protein load (2.5 g/kg; SAVAS whey protein 100, Meiji, Japan), and blood samples were collected at indicated time points for the measurement of plasma branched‐chain amino acids (BCAA).

### 2.9 BCAA measurement

Plasma BCAA concentration was measured using an enzymatic spectrophotometric assay as described by Beckett.^14^ Briefly, bacterial leucine dehydrogenase was used to catalyze the oxidation of BCAA, and the production of NADH was measured using a spectrophotometer (excitation 355 nm, emission 460 nm). L‐leucine was used to generate a standard curve to determine sample concentrations.

### 2.10 Statistical analysis

Statistical significance was first analyzed using Bartlett's test for homogeneity of variances, followed by the Williams’ test for dose–dependent studies. The Williams’ test was performed using a one‐tailed significance level of 2.5% (0.025). All data are presented as means ± standard deviations (SDs). The dose–response data were fitted to a four‐parameter logistic curve using GraphPad Prism ver. 5 to determine the half‐maximal inhibitory concentration (IC_50_) values and 95% confidence intervals.

## 3 RESULTS

### 3.1 Development of an enteropeptidase assay system

**Figure 1:**
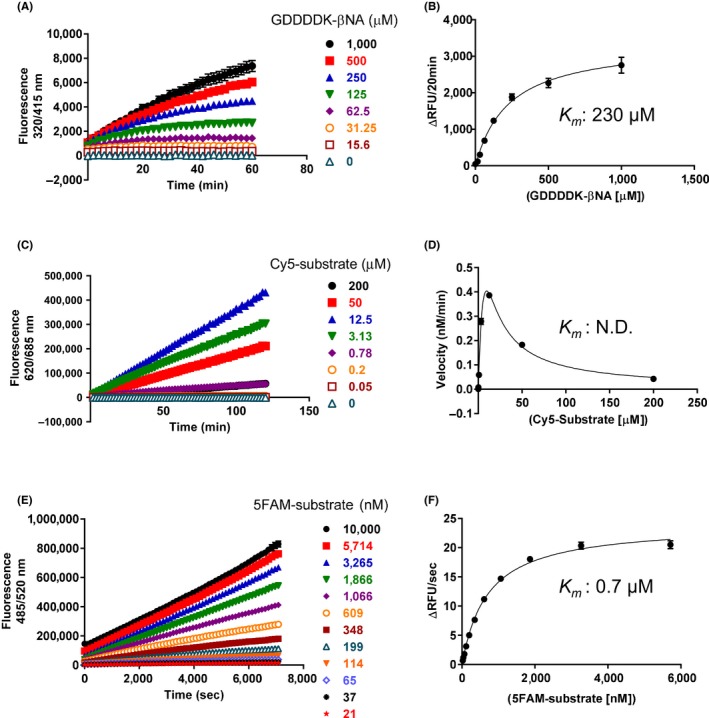
Time course and determination of *K*_*m*_ value of enteropeptidase for various substrates. Enteropeptidase and various concentrations of GDDDDK‐βNA (A), Cy5‐substrate (C), and 5FAM‐substrate (E) were incubated in enteropeptidase assay buffer in a 384‐well plate at room temperature, and the fluorescence was measured. The final concentrations of enteropeptidase were 10 U/mL for GDDDDK‐βNA, 31.3 mU/mL for Cy5 substrate, and 8 mU/mL for 5FAM substrate. Data are presented as mean ± SE values (n = 4). Initial velocities from (A, C, E) are plotted in (B, D, F) as functions of substrate concentration. The data were analyzed using the Michaelis–Menten equation to give *K*_*m*_ values

Enteropeptidase is produced as a proenteropeptidase in enterocytes, the activation of which requires digestion to a light chain and a heavy chain. The light chain contains the catalytic active site, whereas the disulfide‐linked heavy chain provides the anchor to the brush border membrane. Thus, we used recombinant light chain of human enteropeptidase to discover its inhibitors. To identify small‐molecule enteropeptidase inhibitors, we designed two specific dual‐labeled substrates and developed enzyme assay systems. In the assay, the energy of the donor of an uncleaved substrate is transferred to the proximal acceptor at the opposite side of the substrate. When protease cleaves the substrate, the pair separates and the donor no longer transfers its emission to the acceptor, which leads to an increase in the fluorescence intensity observed from the donor.^15^ Enteropeptidase activity was measured with our designed substrates, Cy5‐labeled (Figure 1C) and 5FAM‐labeled substrates (Figure 1E), and commercially available substrate, H‐Gly‐Asp‐Asp‐Asp‐Asp‐Lys‐βNA (GDDDDK‐βNA), naphthylamine (Figure 1A). In our assay system, the fluorescence signal from the enzyme reaction increased in a time‐dependent manner. The calculated *K*_m_ value for the 5FAM‐labeled substrate was 0.7 μmol/L (Figure 1F), which is almost 300 times lower than that of a commercially available substrate, GDDDDK‐βNA, whose *K*_*m*_ value was 230 μmol/L (Figure 1B). Regarding Cy5‐labeled substrate, we could not calculate the *K*_m_ value because the enzyme activity was suppressed at a high concentration of substrate (Figure 1D), which may have been caused by energy transfer not only intramolecularly but also intermolecularly at a high concentration.

### 3.2 Identification of small‐molecule inhibitors of enteropeptidase

**Figure 2:**
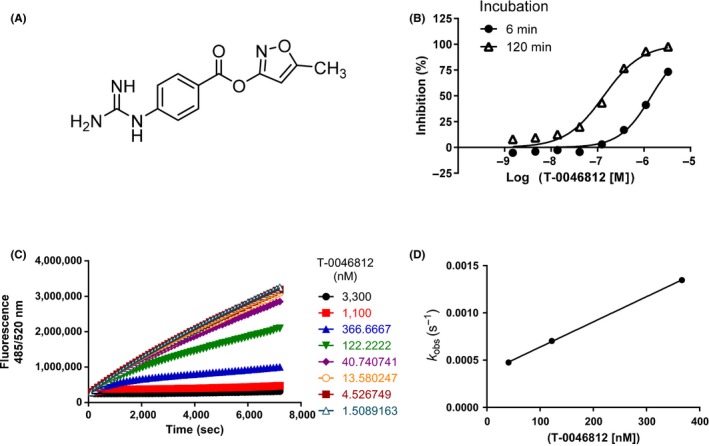
Time‐dependent inhibition of enteropeptidase by the hit compound T‐0046812. (A) Chemical structure of T‐0046812. (B) Percent inhibition is plotted as a function of inhibitor concentration at 6 and 120 minutes of incubation. (C) Enzyme progress curves were obtained at eight different inhibitor concentrations. (D) Data from (C) were fit to Equation to determine observed rate constants (*K*_obs_). Replotting of *K*_obs_ according to Equation yielded *K*_inact_/*K*_*I*_. The estimated *K*_inact_/*K*_*I*_ was 5300 (mol/L s)

We searched for compounds harboring an amidine or guanidine moiety in Takeda's compound library because such compounds are supposed to be good binders to proteases, such as enteropeptidase, that cleave after a basic amino acid residue. As a result, 1116 compounds were screened by enzyme assay using Cy5‐labeled substrate. Among these compounds, 164 that showed >45% inhibition at 30 μmol/L were selected as candidates for enteropeptidase inhibitors. Next, the compounds that met the following conditions were removed: (a) those with no reproducibility of the enteropeptidase inhibition, (b) those with no inhibitory activity in the presence of 10 mmol/L CaCl_2_ (to eliminate chelator‐like inhibitors), and (c) those with > 20% inhibitory activity against human renin at 30 μmol/L. A concentration‐dependent assay was performed on the remaining compounds, 50 of which were selected as hit compounds. The final hit rate for the screen was 4.5%. The most potent hit compound, T‐0046812 (Figure 2A), inhibited enteropeptidase activity with an IC_50_ value of 140 nmol/L (Figure 2B) at 120‐minute incubation. Further kinetic study revealed that the inhibition by T‐0046812 was time‐dependent (Figure 2B and C), and the *k*_inact_/*K*_*I*_ value was 5300 (/mol/L s) (Figure 2D). The structure of T‐0046812 is expected to undergo hydrolysis at its ester moiety to form a covalent bond with the catalytic serine of enteropeptidase.

### 3.3 Biochemical characterization of the investigated drug, SCO‐792

**Figure 3:**
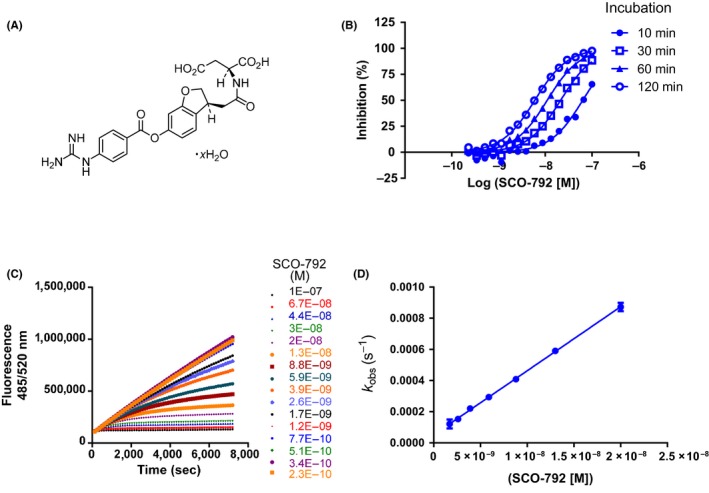
Time‐dependent inhibition of enteropeptidase by SCO‐792. (A) Chemical structure of SCO‐792. (B) Percent inhibition is plotted as a function of inhibitor concentration at 10, 30, 60, and 120 minutes of incubation. (C) Enzyme progress curves were obtained at 16 different inhibitor concentrations. (D) Data from (C) were fit to Equation to determine observed rate constants (*K*_obs_). Replotting of *K*_obs_ according to Equation yielded *K*_inact_/*K*_*I*_. The estimated *K*_inact_/*K*_*I*_ was 82 000 (/mol/L s)

In the optimization process, we improved the chemical stability by conversion of the isoxazole ring of T‐0046812 because the compound is unstable at pH 1.2 and 6.8. Moreover, a polar group was introduced to T‐0046812 for potency and to achieve low absorption. Optimization through ligand‐based drug design of this hit compound from HTS led to the discovery of SCO‐792 (Figure 3A), which showed strong inhibitory activity against enteropeptidase with IC_50_ values of 4.6 and 5.4 nmol/L in rats and humans, respectively (Table 1). Further kinetic study revealed that the inhibition of enteropeptidase by SCO‐792 was time‐dependent (Figure 3B and C) and the *k*_inact_/*K*_*I*_ value was 82 000 (mol/L s) (Figure 3D).

**Table 1:** IC_50_ values of SCO‐792 for human and rat enteropeptidase activities following 120‐minute incubation

	IC_50_ (nmol/L)
	(95% confidence intervals)
Human	5.4 (4.7–6.1)
Rat	4.6 (4.2–5.0)

**Figure 4:**
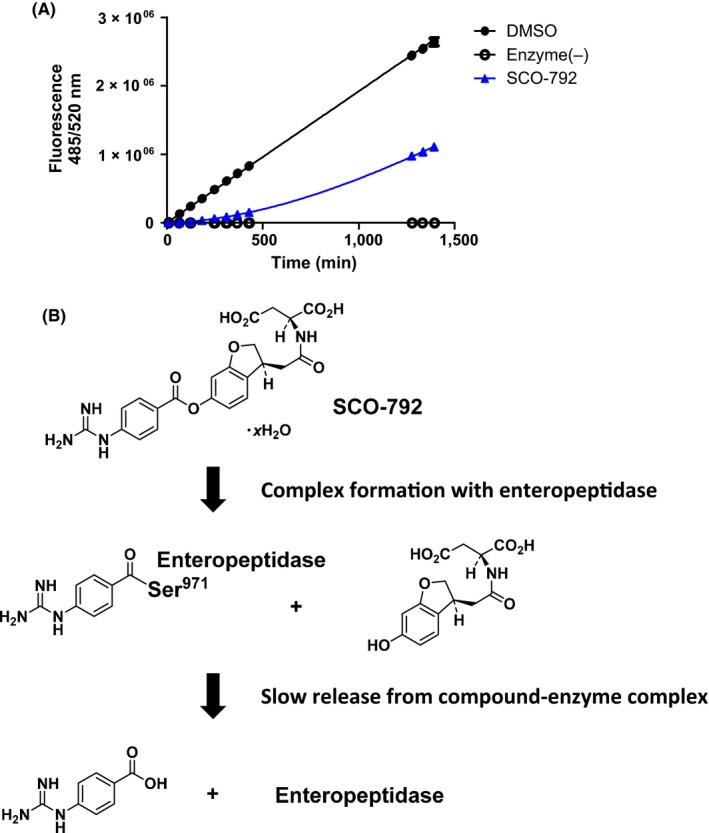
Determination of dissociation half‐life and predicted molecular mechanism of SCO‐792. (A) Enteropeptidase was preincubated for 120 min with and without SCO‐792 and diluted rapidly with substrate solution. Recovery of enteropeptidase activity was detected kinetically. SCO‐792 exhibits very slow reversibility with an estimated *t*_1/2_ value of approximately 14 hours. (B) Predicted mechanism of the inhibitory and reversible activity of SCO‐792

A dissociation assay revealed that inhibition by SCO‐792 was reversible and release of the compound from the enteropeptidase–compound complex should occur very slowly, with a calculated dissociation *t*_1/2_ and *K*_off_ rate of 14 hour and 0.047/hour (Figure 4A). Based on the structure of SCO‐792, the ester moiety of the compound is expected to be subjected to quick nucleophilic attack by the catalytic serine of enteropeptidase; moreover, based on the result of the dissociation assay, the covalent complex of guanidinobenzoate and enteropeptidase is expected to be hydrolyzed slowly after the formation of covalent bonds (Figure 4B). Because of the slowly reversible nature, SCO‐792 is considered to exhibit the desired activity at a sufficient level in vivo.

Regarding selectivity against other serine proteases, SCO‐792 showed < 50% inhibitory activity against chymotrypsin, DPP‐4, factor XIIa, factor Xa, and thrombin at 10 μmol/L. Additionally, SCO‐792 inhibited plasma kallikrein with an IC_50_ value of 16 nmol/L and plasmin with an IC_50_ value of 460 nmol/L (Panlabs Enzymatic Assay Services, Eurofins). SCO‐792 also inhibited trypsin with an IC_50_ value of 3.3 nmol/L.

### 3.4 Pharmacokinetic profiles of SCO‐792 in rats

**Figure 5:**
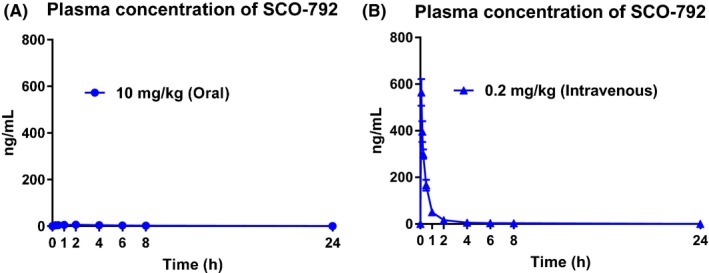
Plasma concentration of SCO‐792 after oral (A) or intravenous (B) administration in rats. SCO‐792 was administrated at the dose of 10 mg/kg orally and 0.2 mg/kg intravenously. Values are presented as the mean ± SD (n = 3). Calculated pharmacokinetic parameters are shown in Table 2

After oral administration of SCO‐792 to rats at a dose of 10 mg/kg, the plasma concentrations of SCO‐792 reached 6.60 ng/mL (*C*_max_) at 1.7 hour (*T*_max_). The *t*_1/2_ and AUC_0‐24 h_ of SCO‐792 were 4.4 hour and 54.1 ng h/mL, respectively (Figure 5A). After intravenous administration of SCO‐792 to rats at a dose of 0.2 mg/kg, the C_5 min_ of SCO‐792 was 564 ng/mL. The *t*_1/2_ and AUC_0‐24 h_ of SCO‐792 were 5.2 hour and 303 ng h/mL, respectively (Figure 5B). The bioavailability (BA) of SCO‐792 calculated from the AUC_0‐24 h_ after oral administration at a dose of 10 mg/kg and that after intravenous administration at a dose of 0.2 mg/kg was 0.4% (Table 2).

**Table 2:** Pharmacokinetic parameters

Admin. route	Dose (mg/kg)	Pharmacokinetic parameters							
		*C*_max_ (ng/mL)	*T*_max_ (h)	*T*_1/2_ (h)	AUC_0–24 h_ (ng h/mL)	AUC_inf_ (ng h/mL)	Vss (mL/kg)	CL_p_ (mL/h/kg)	BA (%)
Oral	10	6.60 ± 1.36	1.7 ± 0.6	4.4 ± 0.5	54.1 ± 7.5	49.8 ± 5.4	–	–	0.4
Intravenous	0.2	564 ± 58	–	5.2 ± 1.8	303 ± 23	304 ± 30	1290 ± 299	663 ± 66	

*C*_max_ after intravenous dosing was observed at 5 minutes. Values are presented as the mean ± SD (n = 3). *C*_max_, maximum plasma concentration; *T*_max_, time to reach *C*_max_; *T*_1/2_, elimination half‐life; AUC_0‐24 h_, area under the plasma concentration vs time curve from 0 to 24 hour after administration; AUC_inf_, area under the plasma concentration vs time curve from 0 to infinity after administration; Vss, volume of distribution at steady state; CLp, plasma clearance; BA, bioavailability.

### 3.5 In vivo inhibition of enteropeptidase in rats

**Figure 6:**
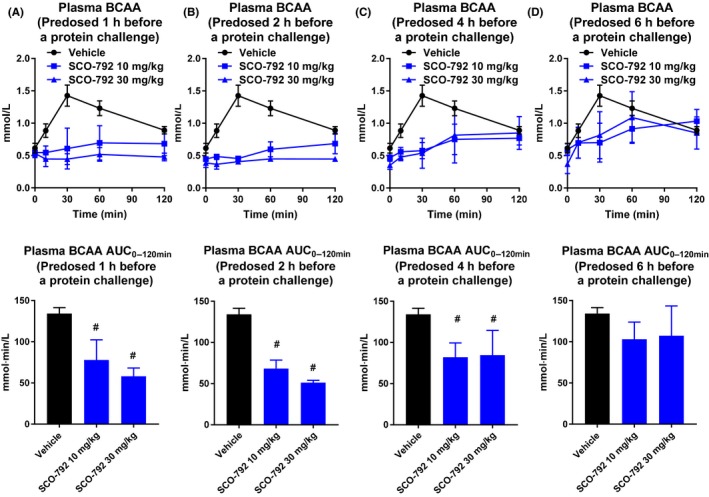
Effect of a single oral dose of SCO‐792 on plasma BCAA levels after an oral protein challenge in rats. Plasma BCAA levels at 1 (A), 2 (B), 4 (C), and 6 hours (D) after oral SCO‐792 dosing in an oral protein challenge test. SCO‐792 effectively suppressed BCAA elevation in this study. Values are presented as the mean ± SD (n = 4). ^#^ *P* ≤ 0.025 vs vehicle by one‐tailed Williams’ test

Given that SCO‐792 was found to be a potent inhibitor against duodenal enteropeptidase activity, which is essential for dietary protein digestion and absorption, inhibiting this enzyme should result in inhibition of gut protein digestion and amino acid absorption. Thus, in vivo effects of oral administration of SCO‐792 on plasma BCAA levels (a reflection of the gut's absorption of digested protein manifested in blood circulation) were tested in rats. This study revealed that a ≤4 hour prior oral dosing of SCO‐792 effectively and dose‐dependently inhibited plasma BCAA elevations induced by oral protein dosing in rats (Figure 6).

## 4 DISCUSSION AND CONCLUSIONS

In this study, we reported the discovery and pharmacological profiles of SCO‐792, a novel inhibitor of enteropeptidase. Our screen using a novel substrate identified a series of enteropeptidase inhibitors, and compound optimization resulted in identification of SCO‐792, which potently and time‐dependently inhibited enteropeptidase activity in vitro. SCO‐792 also showed a slow dissociation property against enteropeptidase in vitro. When tested in vivo, oral dosing of SCO‐792 effectively inhibited gut protein absorption. These results indicate that SCO‐792 is a potent in vitro and in vivo inhibitor against enteropeptidase.

The most common way to detect protease activity is based on specifically labeled substrates.^15^ In this approach, the cleaved substrates contain a chromophore or fluorophore, which enables detection of the signal of the product. There are commercially available chromophore‐ or fluorophore‐added substrates for enteropeptidase; however, for some of them, signal interference occurs because of the intrinsic color or fluorescence of the compound. Moreover, the chromophores and fluorophores used in commercially available substrates are not very sensitive. To develop a sensitive and robust enteropeptidase assay, we designed novel substrates containing fluorophores and corresponding quenchers at either end of peptides in which the quencher absorbs light energy from the fluorophore in an uncleaved state. We used cyanine Cy5 dye as a donor and QSY21 as an acceptor for conducting HTS, and since the emission wavelength of Cy5 is longer than that for the usual sources from the compound itself, a Cy5‐labeled substrate is less susceptible to false‐positive/negative results. We also developed a system using CPQ2 and 5FAM in combination for a long assay period because Cy5 is subjected to oxidation by environmental ozone or other oxidants, which results in a decrease in fluorescence intensity.^16^ Moreover, both Cy5‐ and 5FAM‐labeled substrates cover both prime and non‐prime sides, which enable us to evaluate both prime side‐binding and non‐prime side‐binding inhibitors. In addition, the new method using a Cy5‐labeled or 5FAM‐labeled substrate requires only 30 mU/mL or 8 mU/mL of enzyme each, which corresponds to approximately 0.3% or 0.08%, respectively. By comparison, the assay using GDDDDK‐βNA needs a large amount of enzyme (typically 10 U/mL). As a result, we achieved a reduction of enzyme concentration in the assay, so we could evaluate stronger inhibitors kinetically using the highly sensitive substrates.^17^

Enteropeptidase is a specific protease that recognizes and cleaves after a basic amino acid residue.^18^ Therefore, we screened compounds consisting of an amidine or guanidine moiety because the compounds with amidine or guanidine are known to inhibit proteases that cleave after a basic amino acid residue.^19^, ^20^ Successful identification of a lead compound and optimization process resulted in the discovery of SCO‐792. The present study demonstrated that SCO‐792 was highly effective in inhibiting enteropeptidase both in vitro and in vivo. Many proteases, such as factor Xa, thrombin, plasma kallikrein, plasmin, and trypsin preferentially cleave after a basic amino acid residue.^21^, ^22^ An in vitro selectivity assay revealed that SCO‐792 did not inhibit factor Xa and thrombin. In contrast, SCO‐792 inhibited plasma kallikrein, plasmin, and trypsin in vitro, with IC_50_ values of 16 nmol/L, 460 nmol/L, and 3.3 nmol/L, respectively. Pharmacokinetic analysis revealed very low BA (0.4%) of SCO‐792 in rats, which was consistent with observations reporting that chemical inhibitors containing a highly basic amidine or guanidine group were poorly absorbed into the systemic circulation.^23^, ^24^ Considering the observation that plasma kallikrein and plasmin present in blood plasma exert enzymatic activities,^25^, ^26^ SCO‐792 is unlikely to inhibit circulating kallikrein and plasmin in vivo, and low plasma exposure of SCO‐792 may mitigate unexpected toxic side effects. SCO‐792 also inhibited trypsin activity in vitro. As described in the introduction, trypsin is a key downstream molecule of enteropeptidase and thereby mediates protein degradation.^5^, ^6^ Taken together with the fact that trypsin itself mediates trypsinogen activation (autoactivation),^27^, ^28^ SCO‐792‐induced trypsin inhibition may have contributed to the observed in vivo efficacy.

Enteropeptidase exists on the brush border membrane of the intestine. This means that an enteropeptidase inhibitor has to reach the brush border area of the gut to effectively inhibit enteropeptidase activity. When tested in rats, prior oral administration of SCO‐792 effectively inhibited the elevation of plasma BCAA in an oral protein challenge test, which indicated that SCO‐792 inhibited protein digestion and subsequent absorption in vivo. In the process of compound optimization, we noticed that slow dissociation of compound from enteropeptidase is very important for inhibition of enteropeptidase activity in vivo. In fact, the drug–target residence time has been reported to be an important parameter for in vivo efficacy.^29^, ^30^ In particular, the importance of drug‐residence time for enteropeptidase inhibitors probably depends on their mechanism of action site. Since enteropeptidase inhibitors act in the intestinal tract without circulating in the blood, compounds have a limited chance to interact with enteropeptidase inside the intestinal tract. Thus, slow dissociation of SCO‐792 with enteropeptidase probably contributes to the duration of in vivo efficacy.

Recently, in vivo enteropeptidase inhibition by SCO‐792 was demonstrated to be highly effective in improving the disease status of diabetes and obesity in mouse models.^31^ In addition, inhibition of intestinal trypsin, which is a downstream molecule of enteropeptidase, was shown to decrease body weight and improve metabolism in leptin‐deficient and DIO mice.^32^ Taken these together, protein digestion inside the gut likely to have a significant role in regulating body weight and metabolism and inhibiting this step may be a rational strategy to improve obesity and diabetes.

SCO‐792 showed time‐dependent inhibition, which is a characteristic of covalent inhibitors. Moreover, based on the structure of SCO‐792, the compound is expected to covalently bind to the active site of enteropeptidase, and the covalent bond that forms between the compound and enteropeptidase is expected to be hydrolyzed, as observed for orlistat,^33^ which is a reversible covalent lipase inhibitor. Although covalent drugs have generally been avoided as a pharmaceutical approach because of concerns about side effects, there are many examples of effective and FDA‐approved drugs that act through covalent mechanisms.^34^ Covalent inhibitors react with their target proteins to form a covalent complex, and the protein loses its function. Covalent inhibitors have the advantage of high potency and longer duration of action, and many drugs, such as those for EGFR, BTK, and MetAP2 inhibition, have progressed to Phase II or III clinical trials with acceptable side effects. Additionally, reversible covalent inhibitors are attracting attention because they exhibit sufficiently long efficacy without the potential for side effects resulting from permanent bonding between the compound and target protein.^35^ Accordingly, the reversible covalent inhibitor character of SCO‐792 has an advantage for demonstrating in vivo biological effects because such a compound effectively inhibits the proteolysis of trypsinogen and following digestive signals with reduced side effects over long time periods.

In summary, our new screen identified SCO‐792 as a potent and reversible enteropeptidase inhibitor against enteropeptidase. SCO‐792 showed slow dissociation against enteropeptidase in vitro. When orally dosed to rats, SCO‐792 effectively inhibited protein digestion. Taken together, SCO‐792 was found to be an effective enteropeptidase inhibitor in vitro and in vivo. Further study using SCO‐792 could demonstrate the effects of inhibiting enteropeptidase on biological actions.

## CONFLICT OF INTEREST

This study was conducted with the financial support of Takeda Pharmaceutical Company, Ltd. Among the authors, M.S., I.M., S.I., H.Y., H.H., K.T., K.H., Y.M., M.W., K.T., M.S., J.S., and T.K. are/were employees of Takeda Pharmaceutical Company Ltd., and Y.M. and M.W. are employees of SCOHIA PHARMA, Inc.

## AUTHOR CONTRIBUTIONS

Participated in research design: Masako Sasaki, I. Miyahisa, J. Sakamoto, H. Yashiro, K. Hamagami, M. Watanabe. Designed and analyzed focused library: I. Miyahisa, S. Itono. Conducted experiments: Masako Sasaki, H. Yashiro, H. Hiyoshi, K. Tsuchimori, K. Hamagami, K. Tohyama. Performed data analysis: Masako Sasaki, H. Yashiro, H. Hiyoshi, K. Tsuchimori, K. Hamagami, K. Tohyama, Y. Moritoh. Synthesized the compounds: Minoru Sasaki. Wrote or contributed to the writing of the manuscript: Masako Sasaki, Y. Moritoh, M. Watanabe, T. Kawamoto.

## DATA ACCESSIBILITY STATEMENT

The data that support the findings of this study are available from the corresponding author upon reasonable request.
